# cGAS-STING pathway in pathogenesis and treatment of osteoarthritis and rheumatoid arthritis

**DOI:** 10.3389/fimmu.2024.1384372

**Published:** 2024-05-03

**Authors:** XiCheng Yang, LiLi Zhao, YinQuan Pang

**Affiliations:** ^1^ Graduate School, Hebei Medical University, Shijiazhuang, Hebei, China; ^2^ Orthopedics and Arthrology, People Hospital of Xingtai, Xingtai, Hebei, China; ^3^ Graduate School, Chengde Medical University, Chengde, Hebei, China

**Keywords:** cGAS/STING, CGAS, STING, arthritis, osteoarthritis, rheumatoid arthritis

## Abstract

Osteoarthritis (OA) and Rheumatoid Arthritis (RA) are significant health concerns with notable prevalence and economic impact. RA, affecting 0.5% to 1.0% of the global population, leads to chronic joint damage and comorbidities. OA, primarily afflicting the elderly, results in joint degradation and severe pain. Both conditions incur substantial healthcare expenses and productivity losses. The cGAS-STING pathway, consisting of cyclic GMP–AMP synthase (cGAS) and stimulator of interferon genes (STING), is a crucial component of mammalian immunity. This pathway is responsible for detecting foreign DNA, particularly double-stranded DNA (dsDNA), triggering innate immune defense responses. When cGAS recognizes dsDNA, it catalyzes the synthesis of cyclic GMP–AMP (cGAMP), which then binds to and activates STING. Activated STING, in turn, initiates downstream signaling events leading to the production of interferons and other immune mediators. The cGAS-STING pathway is essential for defending against viral infections and maintaining cellular balance. Dysregulation of this pathway has been implicated in various inflammatory diseases, including arthritis, making it a target for potential therapeutic interventions. Understanding the intricate molecular signaling network of cGAS-STING in these arthritis forms offers potential avenues for targeted therapies. Addressing these challenges through improved early detection, comprehensive management, and interventions targeting the cGAS-STING pathway is crucial for alleviating the impact of OA and RA on individuals and healthcare systems. This review offers an up-to-date comprehension of the cGAS-STING pathway’s role in the development and therapeutic approaches for these arthritis types.

## 1 Introduction

Rheumatoid arthritis (RA) imposes a significant health burden due to its persistent and widespread impact, affecting about 0.5% to 1.0% of the global population, with a higher prevalence in women. The disease’s chronicity results in ongoing joint damage, deformities, and functional limitations, profoundly affecting patients’ daily lives (1, 2). Its economic implications are noteworthy, covering various healthcare expenses and productivity losses (3). RA is linked to an elevated risk of comorbidities, particularly cardiovascular diseases, complicating the overall management (4). RA is typified by immune dysregulation, manifesting as persistent inflammation and hyperplasia of the synovium, resulting in erosive damage to the joints. In RA, inflammatory mediators, including cytokines and autoantibodies, instigate synovial inflammation, the formation of pannus tissue, and erosion of both cartilage and bone. These inflammatory processes entail intricate interactions between various immune cell populations, cytokines, and signaling pathways, ultimately driving detrimental alterations in joint architecture (5). The condition demands frequent healthcare interactions, intensifying healthcare utilization. Furthermore, RA contributes to mental health challenges, including anxiety and depression, and disrupts personal and social dynamics, potentially leading to social isolation (6). The variability in disease severity and treatment responses introduces complexities in care. Addressing these challenges through improved early detection, effective treatments, and comprehensive management is essential to alleviate the impact of RA on individuals and healthcare systems (7). In contrast to RA, osteoarthritis (OA) is a chronic musculoskeletal disorder in which biomechanical factors play a pivotal role and primarily affect the elderly. This biomechanical damage often stems from factors such as age-related changes (8), environmental factors (9), joint misalignment (10), or excessive mechanical loading (11). Nevertheless, OA manifests through the progressive degradation of cartilage and modifications in joint structures leading to cartilage wear, osteophyte formation, and subchondral bone remodeling (12). The disease results in severe joint pain, stiffness, and reduced mobility, significantly impacting the quality of life for affected individuals. Although biomechanical stress also contributes to joint damage in RA, particularly in later disease stages, it is the inflammatory and proliferative mechanisms that predominantly dictate disease progression (13). On the other hand, OA is not a single clinical entity and includes a spectrum of conditions such as post-traumatic osteoarthritis (14) or calcium pyrophosphate crystal-associated osteoarthritis (CPPD) (15) associated. It should be noted that the inflammatory pathogenesis of these two arthritis is convergent in some way. Recognizing these disparate pathogenic pathways is imperative for tailoring effective treatment approaches (16). A comprehensive understanding of the primary drivers of joint damage in OA and RA enables clinicians to devise tailored interventions aimed at alleviating symptoms and preserving joint function effectively. Osteoarthritis is a complex condition influenced by aging, environmental factors, and biochemical changes. Immune cells, particularly activated neutrophils, macrophages, and T cells, play a pivotal role in driving inflammation in the affected joints. Proinflammatory cytokines such as IL-1β, TNF-α, and IL-6, along with chemokines, contribute to the recruitment of immune cells, amplifying the inflammatory cascade. Matrix metalloproteinases (MMPs), including MMP-1, MMP-3, and MMP-13, contribute to the breakdown of the extracellular matrix in articular cartilage. Signaling pathways such as NF-κB, MAPK, and PI3K/AKT are activated, regulating the expression of inflammatory genes (17). Signaling pathways play pivotal roles in the pathogenesis of RA. Dysregulation of key pathways such as JAK-STAT, MAPK, PI3K-AKT, SYK, Wnt, and Notch contributes to the chronic inflammation, immune system abnormalities, and joint damage observed in RA. Abnormal activation of these pathways in synovial cells, immune cells, and cartilage is associated with increased production of pro-inflammatory cytokines, hyperplasia of synovial tissues, and bone destruction. Transcription factors like NF-κB, along with epigenetic modifications, further amplify the inflammatory response. Understanding the intricate molecular signaling network in RA provides valuable insights for developing targeted therapies to modulate these pathways and alleviate disease symptoms (18). The cyclic GMP–AMP synthase (cGAS)–stimulator of interferon genes (STING) pathway plays a pivotal role in mammalian immunity by detecting foreign DNA, particularly double-stranded DNA (dsDNA), and initiating innate immune defense responses. Moreover, the cGAS–STING pathway is connected to NF-κB-mediated transcriptional activation, autophagy, responses that inhibit proliferation, and cell death. These associations contribute to its involvement in both antiviral defense and maintaining cellular balance. Irregularities in this pathway are linked to inflammatory diseases, prompting ongoing initiatives to devise therapeutic approaches targeting cGAS–STING for diverse conditions, especially arthritis (19). There is ample evidence demonstrating the dysregulation of the cGAS-STING signaling pathway implicated in the pathogenesis of various types of arthritis, especially OA and RA. Despite the distinct and multifactorial pathogeneses of OA and RA, substantial evidence suggests that the dysregulation of the cGAS-STING signaling pathway is implicated in the inflammatory pathogenesis of various types of arthritis, particularly in these two conditions (20, 21). Therefore, this review aims to present the current understanding of the role of this pathway in the pathogenesis of OA and RF and provides novel strategies to target cGAS-STING in them.

## 2 cGAS-STING pathway in OA and RA

### 2.1 Overview of cGAS-STING pathway

**Figure 1:**
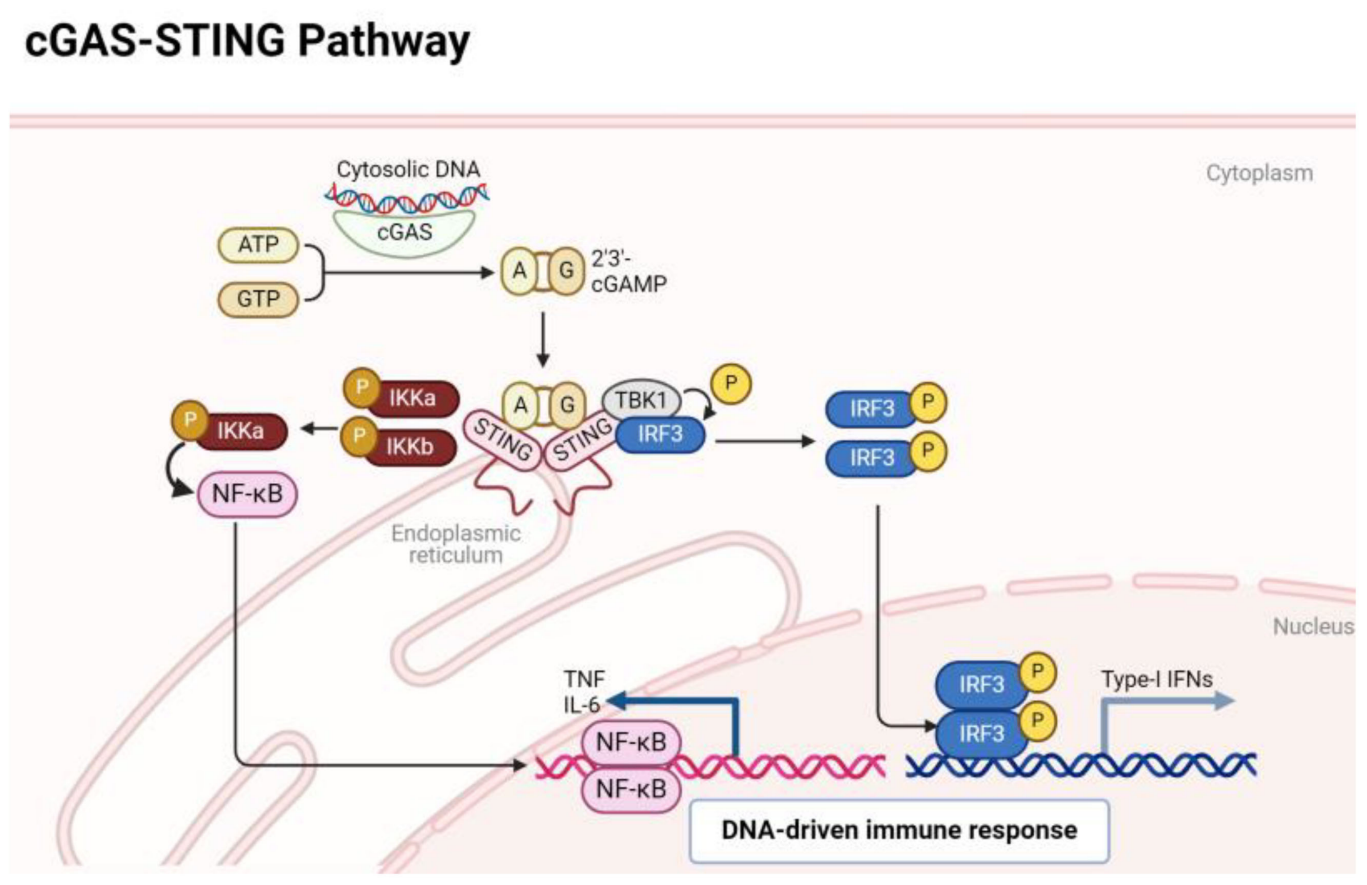
A quick overview of cGAS/STING signaling pathway.

The activation of the cGAS-STING pathway occurs when cytoplasmic DNA is detected, and this DNA can arise from various sources such as viral or bacterial infections, as well as cellular stress or damage. In both RA and OA, genomic mechanisms contribute to the generation of cytoplasmic DNA, often stemming from cellular stress, inflammation, or tissue damage characteristic of these arthritic conditions (5, 12). In RA, aberrant immune responses result in increased cell death, leading to the release of nuclear DNA fragments into the cytoplasm (22). Similarly, in OA, mechanical stress and inflammation in the joint microenvironment can trigger the release of mitochondrial or nuclear DNA (23). The cGAS-STING pathway orchestrates a critical arm of the innate immune system, honing in on the vigilant detection of cytoplasmic DNA (24). At the forefront of this surveillance system is the enzyme cGAS, acting as a sentinel that recognizes and binds to double-stranded DNA. Upon activation, cGAS catalyzes the synthesis of cyclic GMP-AMP (cGAMP), a secondary messenger that sets in motion a cascade of events. The epicenter of this response is the endoplasmic reticulum-bound protein STING, which engages with cGAMP and subsequently recruits the kinase TBK1 (TANK-binding kinase 1). Alternatively, in the absence of a discernible ligand, endoplasmic reticulum stress has been observed to potentially activate the STING. This activation mechanism is believed to occur through calcium and reactive oxygen species (ROS)-mediated mitochondrial impairment, leading to the release of mitochondrial DNA (mtDNA). Hypoxia has the potential to disrupt electron transport and induce mitochondrial damage directly. Nonetheless, it is plausible that disturbances in endoplasmic reticulum calcium balance, increased ROS production, and heightened stress levels could contribute to mitochondrial impairment and subsequent release of mitochondrial DNA (mtDNA) into the cytosol. This release of mtDNA could trigger the cGAS, prompting the production of cGAMP, thereby activating the STING pathway (25, 26). This catalytic action culminates in the phosphorylation of key players such as interferon regulatory factor 3 (IRF3) and IkappaB kinase (IKKs) orchestrating the activation of a DNA immune response. Phosphorylated IRF3 and NF-κB transcription factor translocates to the nucleus, instigating the expression of type I interferons and inflammatory cytokines (19). An overview of these events is depicted in **Figure 1**.

### 2.2 cGAS-STING in pathogenesis of OA and RA

**Figure 2:**
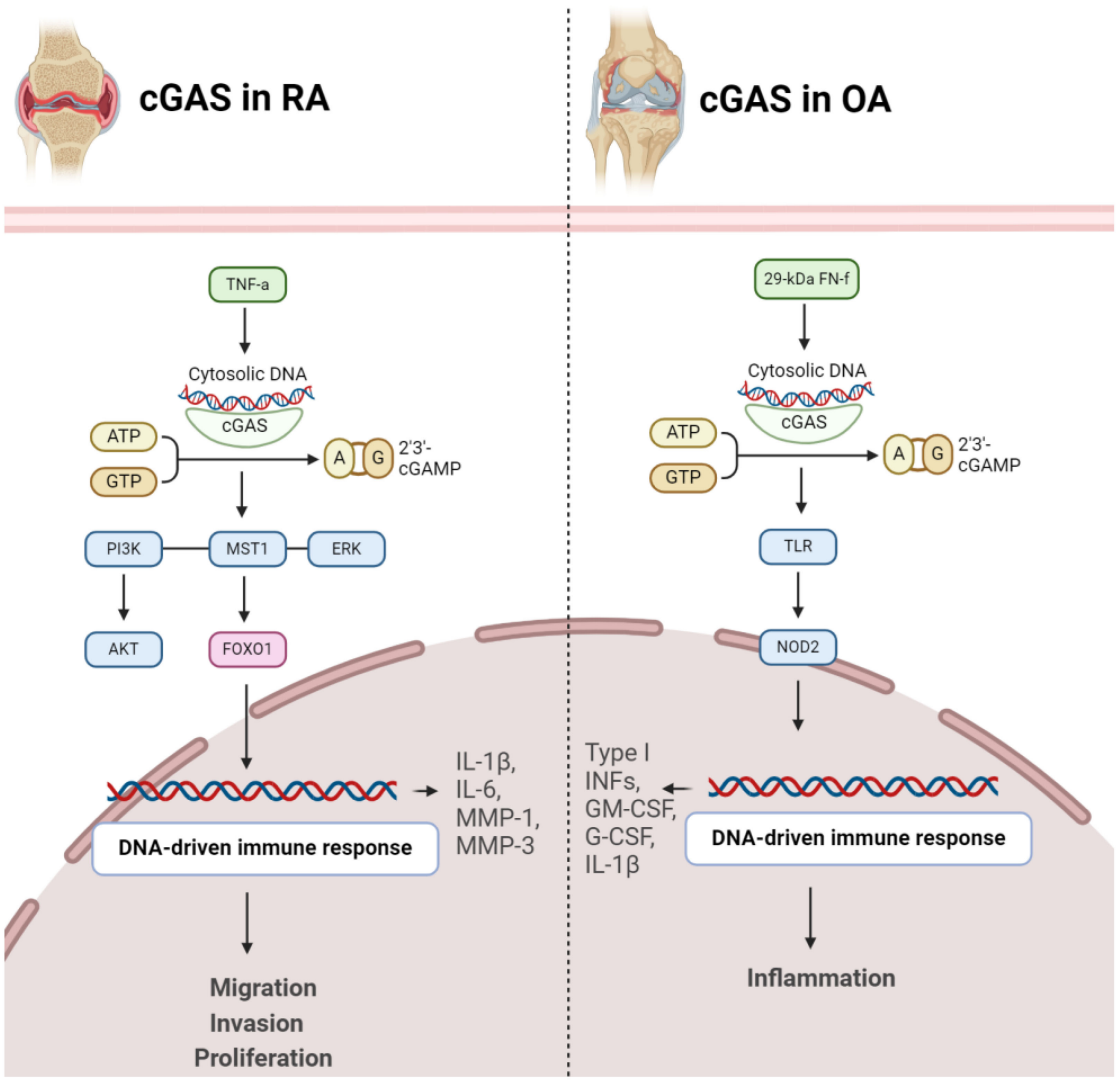
Role of cGAS in pathogenesis of RA (left) and OA (right). In both conditions, activation of cGAS results in disease progression.

The cGAS/STING signaling pathway assumes a pivotal role in regulating the aggressive behavior of rheumatoid synovial tissues. Within the context of RA, fibroblast-like synoviocytes (FLSs) display tumor-like characteristics, influencing persistent joint inflammation and destruction. The accrual of cytosolic double-stranded DNA (dsDNA) in RA FLSs triggers the cGAS/STING pathway, activating STING and prompting the generation of type I interferons. This activation propels the migration and invasion of RA FLSs, crucial factors in the aggressive nature of rheumatoid synovial tissues. Inhibiting cGAS or STING expression curtails dsDNA-induced migration and invasion of RA FLSs, underscoring the pivotal role of the cGAS/STING pathway in shaping the invasive profile of FLSs. Additionally, the role of MST1-FOXO1 pathway was highlighted, elucidating that MST1 activation, influenced by the cGAS/STING pathway, impacts FOXO1 phosphorylation and nuclear translocation. FOXO1 emerges as a critical downstream element of MST1, contributing to the regulatory mechanisms governing dsDNA-induced migration and invasion of RA FLSs, thereby enhancing our comprehension of the intricate signaling dynamics in the pathogenesis of rheumatoid arthritis (**Figure 2**) (21). In inflammatory arthritis, cGAS and STING function as pattern recognition receptors (PRRs). These receptors play a critical role in the innate immune response by detecting cytosolic DNA, particularly during cellular stress or viral infections. In the presence of the pro-inflammatory cytokine TNF, prolonged stimulation leads to cGAS and STING activation, resulting in the release of mitochondrial DNA into the cytosol. This triggers the STING pathway, inducing a type I interferon (IFN) response and the upregulation of interferon-stimulated genes (ISGs). cGAS deficiency ameliorates symptoms in an arthritis model, suggesting that cGAS and STING may be involved in the development and progression of inflammatory arthritis. Targeting these pathways could potentially offer therapeutic strategies for diseases characterized by TNF-mediated inflammation (27). Elevated cytosolic dsDNA is detected in RA FLS, especially when exposed to TNF-α, and is linked to the intensity of synovial inflammation. Introducing transfected cytosolic dsDNA induces the generation of inflammatory cytokines and matrix metalloproteinases by RA FLS. RA FLS exhibit diminished DNaseII levels, responsible for cytosolic dsDNA degradation, indicating a possible connection between reduced DNaseII and heightened cytosolic dsDNA. Thus, cGAS/STING pathway is linked with inflammatory reactions triggered by cytosolic dsDNA in RA FLS. The cGAS/STING pathway contributes to inflammatory responses in RA FLS, while DNaseII serves as a regulatory factor controlling cytosolic dsDNA levels and subsequent cytokine production (28).

### 2.3 Role of cGAS in arthritis

#### 2.3.1 cGAS in RA

cGAS emerges as a pivotal contributor to RA development. The expression of cGAS is notably higher in RA-FLS compared to OA-FLS. Stimulation with the proinflammatory cytokine TNFα further escalates cGAS expression over time. Significantly, increased cGAS levels in RA FLS are associated with heightened cell proliferation, suggesting a role in the pathological advancement of RA. Moreover, cGAS facilitates proinflammatory responses by augmenting the production of key mediators (IL-1β, IL-6, MMP-1, MMP-3) in response to TNFα, indicating its involvement in the regulation of inflammatory pathways. Mechanistically, cGAS activation appears to impact AKT and ERK pathways, providing insights into the molecular processes underlying its influence on RA pathogenesis. Conversely, suppressing cGAS mitigates these effects, suggesting a potential therapeutic approach to alleviate inflammation and proliferation in RA. These findings offer a nuanced understanding of cGAS’s intricate role in RA and propose it as a viable target for therapeutic intervention in autoimmune arthritis (29). RA-FLSs and macrophages play a significant role in promoting inflammatory and bone-erosive factors within the synovium (30). Extracellular traps (ETs), such as neutrophil extracellular traps (NETs) and macrophage extracellular traps (METs), consisting of histones, DNA, and other components, have been associated with autoimmune disorders. The accumulation of macrophages and METs, characterized by citrullinated histones (CitH3), in RA synovial tissues has been discovered. METs are also identified in RA synovial fluids. In laboratory experiments, RA-FLSs treated with METs display heightened tumor-like behaviors, including increased proliferation, migration, invasion, and the expression of proinflammatory cytokines and matrix-degrading enzymes. The cGAS pathway is implicated in MET-induced effects, and inhibition of cGAS reverses these responses. Additionally, METs activate the PI3K/Akt signaling pathway in RA-FLSs, connecting the DNA-sensing pathway to downstream signaling involved in tumor-like characteristics. Therefore, METs contribute to the promotion of pathological features in RA-FLSs through the cGAS/PI3K/Akt pathway, offering insights into potential therapeutic targets for treating RA (**Figure 2**) (31).

#### 2.3.2 cGAS in OA

Fibronectin fragments, specifically the 29-kDa fibronectin fragment (29-kDa FN-f), are protein components derived from the breakdown of fibronectin, a large glycoprotein involved in the extracellular matrix of connective tissues. In OA, the effects of 29-kDa FN-f on chondrocytes, the cells embedded in cartilage tissues, have been explored. Exposure to 29-kDa FN-f induces DNA damage in chondrocytes and activates cGAS and STING pathway. This activation leads to the expression of pro-inflammatory cytokines, including type I interferons, granulocyte-macrophage colony-stimulating factor (GM-CSF), granulocyte colony-stimulating factor (G-CSF), and interleukin-1β (IL-1β). Notably, these effects are mediated through Toll-like receptor-2 (TLR-2) and Nucleotide-binding oligomerization domain-containing protein 2 (NOD2) signaling pathways, as silencing of TLR-2 or NOD2 suppresses 29-kDa FN-f-induced activation of the cGAS/STING pathway (**Figure 2**) (32).

### 2.4 Role of STING in arthritis

#### 2.4.1 STING in OA

**Figure 3:**
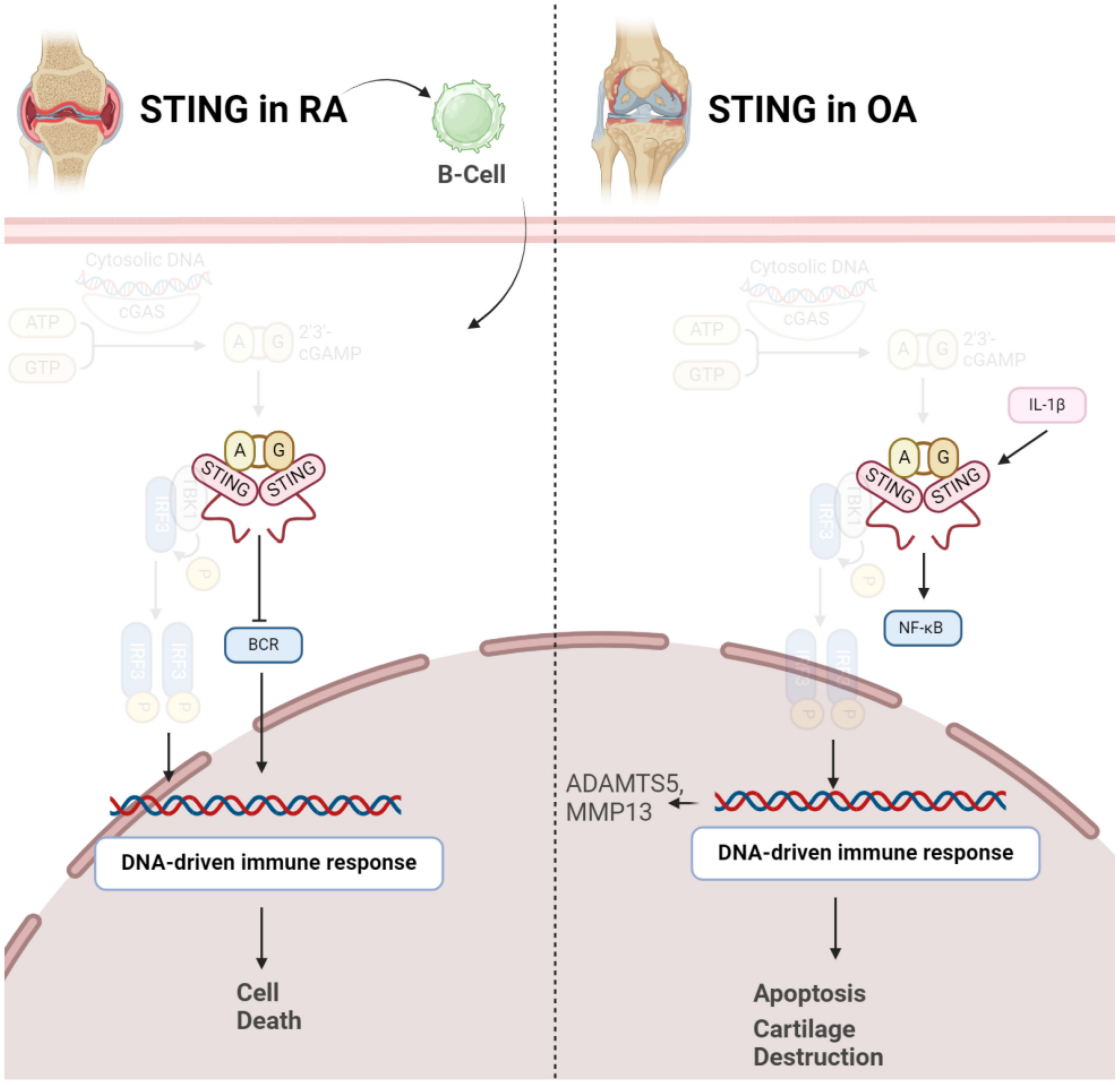
Role of STING in pathogenesis of RA (left) and OA (right). In both conditions, activation of STING results in disease progression via inducing cell death.

STING emerges as a key player in pathogenesis of OA. Elevated STING expression is observed in both human and mouse OA articular cartilage, and its levels increase in response to the pro-inflammatory cytokine IL-1β. STING activation is associated with DNA damage, and its overexpression in chondrocytes induces extracellular matrix (ECM) degradation through the NF-κB signaling pathway. This degradation involves upregulation of matrix-degrading enzymes (ADAMTS5, MMP13) and downregulation of ECM proteins (Collagen II, Aggrecan). Additionally, STING promotes cellular senescence and apoptosis in chondrocytes, processes integral to OA pathology. Importantly, therapeutic interventions, such as STING knockdown, demonstrate promising outcomes in mitigating OA progression in mice, suggesting STING as a potential target for OA treatment by addressing its impact on ECM integrity, cellular function, and disease severity (**Figure 3**) (33). STING plays a crucial role in influencing various aspects of OA. Initially, in chondrocytes exhibiting OA-like characteristics under inflammatory conditions, both STING and IFI204, pattern recognition receptors sensing cytosolic DNA, are specifically upregulated. This heightened expression is associated with catabolic factors like IL-1β, hypoxia-inducible factor (HIF)-2α, or ZIP8, indicating their involvement in OA pathogenesis. Moreover, STING activation is linked to DNA damage in chondrocytes, suggesting a mechanistic connection between DNA damage response and OA progression. Notably, the absence of STING in mice demonstrated a protective effect against post-traumatic OA, emphasizing its direct impact on cartilage destruction and subchondral bone sclerosis. Additionally, STING deficiency reduced OA-associated mechanical allodynia, indicating its role in influencing pain perception during OA. Conversely, stimulating the STING pathway intensified both OA progression and mechanical sensitivity in mice, particularly affecting cartilage destruction and subchondral bone sclerosis, while osteophyte formation remained unaffected. This dual role of STING, both promoting and mitigating OA manifestations, underscores its complex involvement in the disease process. Furthermore, the study explored STING’s impact on pain-sensitization molecules, revealing that STING deficiency reduced the expression of TRPV1 and CGRP in the synovium and meniscus. These findings suggest that STING’s regulatory influence extends beyond joint structural changes to modulating peripheral sensitization, thereby impacting OA-associated pain (34).

#### 2.4.2 STING in RA

In contrast to OA, it should be noted STING might negatively control RA development in early stages of the disease. A study has investigated the role of STING in the pathogenesis of RA using a collagen-induced arthritis (CIA) mouse model. The synovium in RA involves fibroblast-like synoviocytes and macrophage-like synoviocytes, contributing to inflammation and joint damage. STING’s pivotal role in driving the inflammatory cascade of RA even gets bolder, as indicated that in RA, elevated intracellular levels of STING were observed, whereas in OA synovial fluids (SFs), a predominance of extracellular STING was noted. Dysregulation of both Nrf2 and STING pathways has been implicated in RA pathogenesis. Decreased Nrf2 activity and increased STING activation have been observed in RA patients, correlating with disease severity and joint damage (35). Surprisingly, STING is not necessary for arthritis development in the CIA model. While the arthritis scores were comparable between wild-type and STING-deficient mice, the latter showed higher levels of anti-collagen type 2 antibodies, suggesting a potential negative regulatory role for STING in the early stages of autoimmune arthritis. Gene expression profiling of splenocytes revealed changes related to B cell receptor (BCR) signaling, indicating a potential role of STING in B cell biology. Further experiments demonstrated that STING negatively regulates BCR-induced B cell proliferation and mediates B cell death. The findings suggest a nuanced role for STING in RA, influencing B cell responses and potentially acting as a negative regulator in the early stages of autoimmune arthritis (**Figure 3**) (36).

### 2.5 TBK1

#### 2.5.1 TBK1 in OA

**Figure 4:**
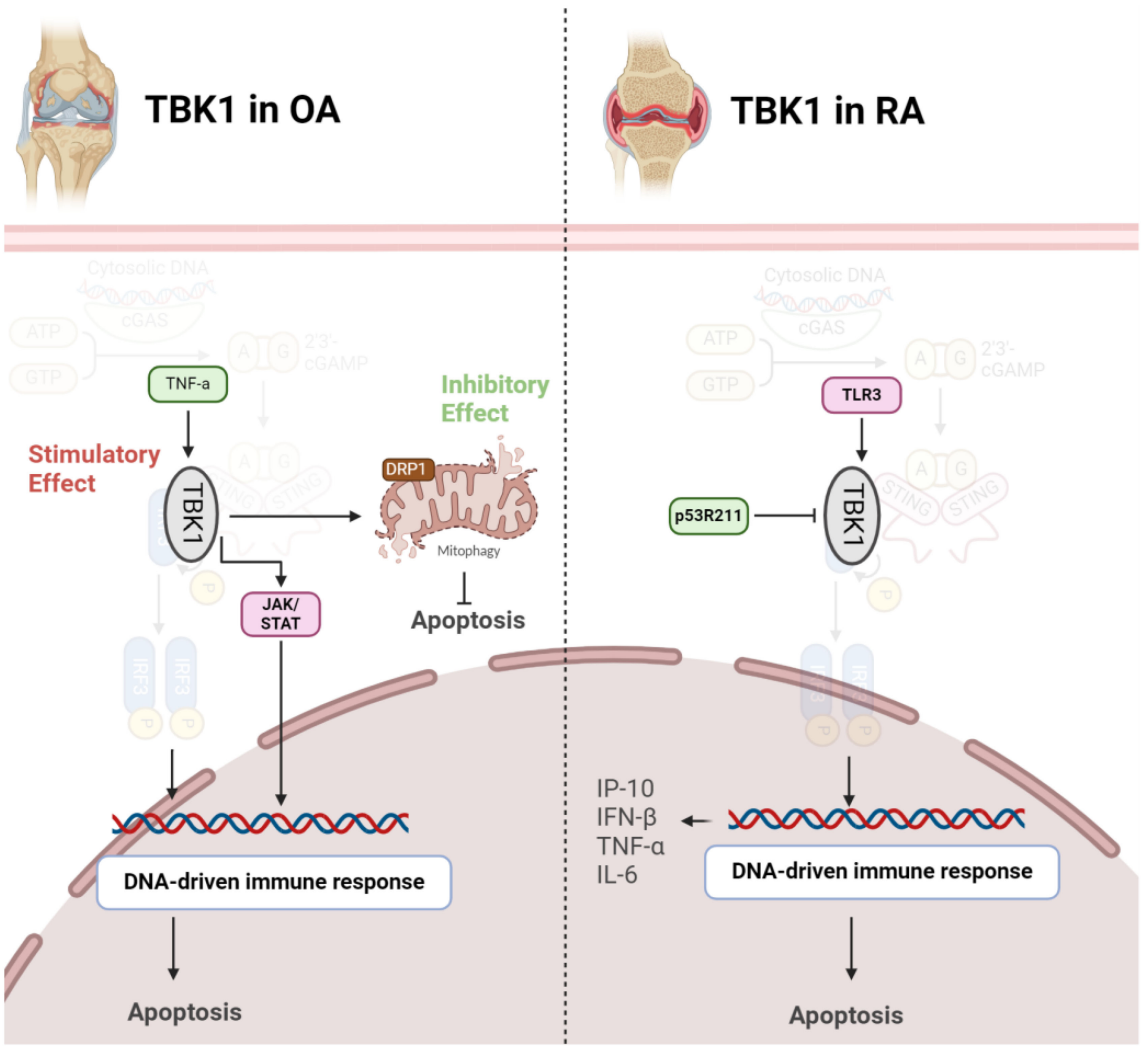
Role of TBK1 in pathogenesis of OA (left) and RA (right). In OA, TBK1 acts as a double-edged sword, inhibiting apoptosis via mitophagy induction or stimulating it through JAK/STAT axis. However, in RA, activation of TBK1 exacerbates diseases by producing inflammatory cytokines.

Several studies have explored the role of TBK1 in OA using animal and cell models. A significant decrease in TBK1 expression in deteriorated cartilage of OA patients and chondrocytes from aged mice has been observed. Upon TNF-α stimulation, there was an elevation in both p-TBK1 S172 and TBK1 proteins. Overexpression of TBK1 in chondrocytes mitigated TNF-α-induced apoptosis, maintained mitochondrial function, and reversed alterations in mitochondrial dynamics. TBK1 directly phosphorylated DRP1 at Ser637, facilitating mitochondrial fusion and hindering apoptosis-related cell death through enhanced mitophagy. Furthermore, TBK1 activation by malvidin-3-O-arabinoside (M3A) during TNF-α stimulation revealed a TBK1-dependent pathway, involving AMPK activation that regulated DRP1 phosphorylation. Intraarticular injection of lentivirus-TBK1 in a destabilization of the medial meniscus (DMM)-induced OA mouse model demonstrated protective effects, suggesting that TBK1 could alleviate OA progression by activating autophagy and suppressing apoptosis. In summary, the findings propose a potential therapeutic function for TBK1 in mitigating the advancement of OA (37). In contrast to the above findings, a study revealed heightened expression of TBK1 in OA models, particularly under interleukin-1 beta (IL-1β) stimulation. Lentivirus-induced TBK1 silencing in OA mice significantly mitigated cartilage degradation, reflected in lowered OARSI scores and diminished serum levels of cartilage degradation markers. Similarly, in an in vitro OA cell model, TBK1 silencing demonstrated a protective impact against ECM degradation. Further analysis indicated that TBK1 activated the JAK/STAT signaling pathway, and the positive effects of TBK1 silencing on ECM degradation were nullified by STAT3 activation (**Figure 4**). These findings emphasize TBK1 as a promising therapeutic target for OA, potentially through its influence on JAK/STAT signaling and modulation of ECM degradation processes (38).

#### 2.5.2 TBK1 in RA

TBK1 assumes a crucial role in modulating the immune response in RA. Upon Toll-like receptor 3 (TLR3) activation in FLS, TBK1 becomes active and contributes to sustained inflammation by phosphorylating interferon regulatory factors (IRFs) 3 and 7. This activation initiates the expression of interferon response genes, particularly C-X-C motif ligand 10 (CXCL10)/IFN-γ-induced protein 10 kDa (IP-10), known for its involvement in RA-related inflammation. TBK1 deficiency results in a notable decrease in IP-10 production, a chemokine associated with RA pathogenesis. Additionally, TBK1 regulates IRF3 activation, IFN-β gene expression, and IP-10 promoter activity in FLS. Consequently, TBK1 stands out as a pivotal element in the molecular pathway contributing to the inflammatory response in RA, presenting itself as a potential target for therapeutic strategies aimed at modulating immune-related mechanisms in this autoimmune disorder (39). TBK1 emerges as a pivotal player in autoimmune diseases and the development of inflammatory arthritis resulting from compromised DNA clearance. In situations where DNase II is lacking, TBK1 becomes recruited to STING, contributing to the onset of polyarthritis. TBK1 activation triggers the heightened production of inflammatory cytokines like TNF-α and IL-6, implicated in joint inflammation and damage. Thus, inhibiting TBK1-mediated signaling, especially its interaction with STING, could serve as a potential therapeutic strategy for alleviating inflammatory arthritis linked to impaired DNA clearance. This understanding of TBK1’s molecular role provides valuable insights into the underlying mechanisms of autoimmune diseases, offering potential targets for therapeutic intervention (22). Mutations are also implicated in pathogenesis of RA. The p53 gene, widely recognized as a tumor suppressor, is involved not just in cancer but also in immune and inflammatory reactions. The p53R211* mutant, a common p53 mutation in cancer, affects RA using an adjuvant-induced arthritis (AIA) rat model. Evidence shows that this mutation has an immunomodulatory effect, alleviating arthritis, reducing synovial hyperplasia, and improving bone destruction. Mechanistically, the mutant inhibits T-cell activation, decreases Th17 cell populations, and suppresses pro-inflammatory cytokines. The anti-arthritic effect involves inactivating immune-related pathways, particularly the TBK1-IRF3-STING signaling cascade. The p53R211* mutant seems to interact with TBK1, inhibiting its activation and downstream immune responses. Therefore, the findings propose a potential therapeutic role for the p53R211* mutant in RA by modulating immune and inflammatory pathways, shedding light on the intricate relationship between p53 mutations and autoimmune diseases (**Figure 4**) (40).

### 2.6 IRF-3

The phosphorylated forms of IKKϵ and IRF-3 were found to be significantly elevated in RA synovial tissue compared to OA tissue. In FLS, stimulation with poly(I-C) led to increased IKKϵ phosphorylation, IRF-3 activation, and elevated IFNβ production. Experiments using a dominant-negative IKKϵ adenovirus demonstrated the crucial role of IKKϵ in poly(I-C)-mediated IRF-3 activation and type I IFN gene expression in FLS. In addition, IKKϵ inhibition suppressed IFNβ and RANTES gene expression at the mRNA level. Findings from IKKϵ−/− mouse synoviocytes further supported the regulatory role of IKKϵ in IFNβ and RANTES gene expression (41). The vital involvement of innate immune responses in cell activation and recruitment within the rheumatoid joint is notable. Recognition of viral and bacterial products by TLRs and innate sensors is thought to contribute to these processes. The gene expression profile in RA synovium indicates exposure to TLR ligands, displaying characteristics of the type I IFN signature. Despite unclear proximal mechanisms, the IκB kinase (IKK)-related kinase, IKKϵ, seems involved in the signaling pathway activated by polyinosinic-polycytidylic acid (poly [I-C])–mediated TLR3 activation in human RA synoviocytes. However, the downstream transcription factors controlling type I IFN-regulated gene expression in RA remain unidentified. It is hypothesized that the IFN regulatory factor (IRF) family, especially IRF3 and IRF7, may regulate the IFN response in RA. The results suggest that, in poly (I-C)–stimulated FLS, IRF3 takes precedence over IRF7 as the primary regulator of type I IFN responses. Unlike many other cell types, primary human RA synoviocytes reveal IRF3 as the dominant transcription factor, with a relatively modest contribution from IRF7. Moreover, IRF3 is found to regulate various cytokines, chemokines, and matrix metalloproteinases (MMPs) through a mechanism involving c-Jun and the AP-1 promoter site (42).

### 2.7 IKK

#### 2.7.1 IKK in OA

Researcher have investigated the distinct roles of IKKα and IKKβ in OA pathogenesis, focusing on their impact on chemokine production by chondrocytes. Utilizing surgical animal models to replicate human OA, the researchers identify key target genes, including interleukin-1β (IL-1β) and ADAMTS5, within the NF-κB pathway. IKKα is found to play a significant role in the loss of maturation arrest of chondrocytes, leading to hypertrophy and terminal differentiation, while chemokines, particularly CCL2/MCP-1, mediate crosstalk between OA cartilage chondrocytes and other joint compartments, contributing to immune cell chemotaxis and synovial inflammation. RNA interference-mediated knockdown of IKKα and IKKβ in chondrocytes reveals their distinct impact on chemokine gene expression, with CCL2/MCP-1 being notably affected. Functional studies demonstrate that conditioned media from chondrocytes with IKKα or IKKβ knockdown inhibit monocyte chemotaxis, emphasizing the critical role of these kinases in OA pathophysiology and suggesting potential therapeutic targets (43). Furthermore, Wang et al. explored the intricate regulatory network involving long non-coding RNAs (lncRNAs), microRNAs (miRNAs), and key signaling pathways in the context of OA. The investigation highlights the role of lncRNA SNHG1 in mitigating OA progression. SNHG1 is identified as a crucial factor in protecting chondrocytes from oxidative stress-induced apoptosis and inflammation. Mechanistically, SNHG1 downregulates miR-195, which, in turn, negatively regulates IKKα—an inhibitor of the NF-κB complex. By employing in vitro experiments using H2O2-treated chondrocytes, it was revealed that SNHG1 overexpression prevents apoptosis, decreases ROS levels, and reduces the production of inflammatory cytokines. Furthermore, SNHG1 is shown to directly target miR-195, and miR-195 is demonstrated to directly target IKKα. By extending these findings to an *in vivo* OA rat model, it was demonstrated that miR-195 antagomiR treatment alleviates OA, accompanied by increased SNHG1 expression and reduced NF-κB signaling. Therefore, SNHG1 may play a protective role in OA by modulating the miR-195/IKKα axis, offering potential therapeutic insights into OA management (44).

### 2.8 Nrf2

The nuclear factor erythroid 2-related factor 2 (Nrf2) is a key transcription factor known for its cytoprotective role against oxidative stress. Recent studies have revealed a complex interplay between Nrf2 and the STING pathway, which plays a critical role in innate immunity and inflammation. Nrf2 has been shown to modulate the activity of the STING pathway through multiple mechanisms. Firstly, Nrf2 activation suppresses the expression of STING and downstream inflammatory cytokines, thereby attenuating the STING-mediated inflammatory response (23, 45). Additionally, Nrf2 activation promotes the expression of antioxidant enzymes such as heme oxygenase-1 (HO-1) and NAD(P)H quinone oxidoreductase 1 (NQO1), which mitigate oxidative stress and dampen STING signaling (46). Furthermore, Nrf2 activation enhances autophagy, a cellular process implicated in the degradation of cytoplasmic DNA and regulation of STING activation. Dysregulation of both Nrf2 and STING pathways has been implicated in RA pathogenesis. Decreased Nrf2 activity and increased STING activation have been observed in RA patients, correlating with disease severity and joint damage (35). Similarly, in OA imbalance in the Nrf2-STING axis contributes to sustained inflammation, cartilage degradation, and joint dysfunction in OA. In this regard the study of Ni and colleagues revealed that The activation of Nrf2 suppresses the STING-dependent NF-κB pathway, consequently mitigating the inflammatory response, extracellular matrix (ECM) degeneration, and senescence observed in chondrocytes when stimulated by IL-1β. Restoring the Nrf2 function or targeting the STING pathway represents promising therapeutic strategies for both OA and RA. Preclinical studies utilizing Nrf2 activators or STING inhibitors have shown efficacy in ameliorating arthritis symptoms and reducing joint inflammation in animal models. Collectively, these findings suggest that Nrf2 serves as a negative regulator of the STING pathway, exerting anti-inflammatory and cytoprotective effects (47).

## 3 cGAS-STING as a Promising Target in Arthritis

Exploring therapeutic interventions that target cGAS/STIN holds significant promise, considering the pivotal role of its downstream targets in the pathogenesis of both osteoarthritis and rheumatoid arthritis. The cGAS/STIN pathway, involved in sensing cytoplasmic DNA, triggers a cascade of events that ultimately lead to the activation of inflammatory responses. The identification of the cGAS-STING pathway as a potential therapeutic target has spurred the development of various strategies to modulate its activity. One approach involves catalytic site inhibitors, which target the enzymatic activity of cGAS, thereby preventing the production of the second messenger molecule, cGAMP, which activates the STING pathway. Examples of such inhibitors include PF-06928125, RU.521, G150, and Compound S3. Another strategy focuses on disrupting the binding of cGAS to double-stranded DNA (dsDNA), which is the activating trigger for the cGAS-STING pathway. Compounds like hydroxychloroquine, quinacrine, suramin, and suppressive oligodeoxynucleotides (e.g., A151) are investigated for this purpose. Additionally, small molecules can be designed to occupy the cyclic dinucleotide (CDN)-binding site of STING, acting as competitive antagonists of STING activators. Examples of such antagonists include tetrahydroisoquinolines and astin C. Furthermore, inhibitors targeting the palmitoylation sites of STING, such as nitrofurans (e.g., C-176, C-178), indole ureas (e.g., H-151), and nitroimidazoles, represent another avenue for modulating the cGAS-STING pathway’s activity. These therapeutic strategies hold promise for the treatment of diseases associated with dysregulated immune responses mediated by the cGAS-STING pathway (19). Therapeutic targeting of the cGAS-STING pathway for the treatment of inflammatory diseases like OA and RA holds promise but comes with potential demerits and challenges that have been investigated. These include the risk of off-target effects, immunosuppression, the complexity of the pathway, disease specificity, potential adverse events, the risk of resistance development, challenges in drug delivery, and limited clinical data. Addressing these challenges will be crucial for ensuring the safety and efficacy of drugs targeting the cGAS-STING pathway in diverse patient populations and disease contexts (48–50). Further research and clinical trials are needed to better understand the therapeutic potential and limitations of targeting this pathway in various diseases.

By strategically addressing cGAS/STIN, researchers aim to modulate the downstream signaling pathways implicated in the progression of osteoarthritis and rheumatoid arthritis. This approach may offer a nuanced and effective means of managing these debilitating joint disorders, potentially providing novel avenues for therapeutic development and improving the quality of life for individuals affected by these conditions. Herein, we introduce different pharmaceutical that can target this pathway in both RA and OA (**Table 1**).

**Table 1:** Therapeutics targeting cGAS/STING pathway in OA and RA.

Therapeutic	Type of Arthritis or inflammatory model	Cell lines	Study type	Targeted	Highlights	
Natural products						
Gelsevirine (GS)	OA	HEK293T	In vitro/In vivo	↓STING ↓MMP3, ↓MMP9, ↓MMP13, ↓IFNβ, ↓TNFα, and ↓Il6 ↑Col2A and ↑Il10.	“GS attenuated the activation of the STING/TBK1 pathway induced by IL-1β, leading to a reversal of the inflammatory response and degeneration in cultured chondrocytes.” “Treatment with GS attenuated the degradation of articular cartilage in both age-related osteoarthritis (OA) and destabilization of the medial meniscus (DMM)-induced OA.”	(51)
Catechin	Collagen-induced arthritis	HEK92	In vitro/In vivo	↓IKK-β, ↓NF-κβ ↓IL-1, ↓IL-6, and ↓IL-17A	“In a mouse model of collagen-induced arthritis, administration of catechin was correlated with diminished paw thickness, decreased clinical scoring, and lowered mRNA levels of inflammatory cytokines.”	(52)
Glabralactone	Carrageenan-induced hind paw edema model	RAW264.7	In vitro/In vivo	↓iNOS, ↓TNF-*α*, ↓IL-1*β*, ↓miR-155, ↓NF-*κ*B, ↓IRF-3	“The anti-inflammatory effects of glabralactone were partially attributed to its ability to suppress the production of proinflammatory mediators, including iNOS and IL-1β, in inflamed paw edema tissues in rat models.” “Glabralactone effectively suppressed the activation of both NF-κB and the TRIF-dependent IRF-3 pathway in macrophages stimulated with LPS.”	(53)
Triptolide	RA	RAW264.7	In vitro/In vivo	↓cGAS-STING ↓TNF-α, ↓IL-1β and ↓IL-6	“In M1 macrophages, the released TP exhibited anti-inflammatory and immunosuppressant properties by down-regulating the expressions of cGAS and STING proteins. Consequently, this down-regulation led to a reduction in the secretion of TNF-α, IL-1β, and IL-6.”	(54)
Synthetic Effectors						
C-176	RA and OA	Bone marrow-derived macrophages (BMMs)	In vitro/In vivo	↓STING, ↓NF-*κ*B, ↓Nuclear factor of activated T-cells c1(NFATc1), ↓cathepsin K, ↓calcitonin receptor, and ↓V-ATPase a3	“C-176 demonstrated the ability to mitigate LPS-induced bone resorption in mice, attenuate joint destruction in knee arthritis induced by meniscal instability, protect against cartilage matrix loss in ankle arthritis induced by collagen immunity and inhibit the activation and formation of osteoclasts.”	(55)
CS12192	RA (adjuvant-induced arthritis, collagen-induced arthritis in rat model)	KHYG-1, UT-7, THP-1, RAW264.7	In vitro/In vivo	↓JAK3/↓JAK1/↓TBK1 ↓STATs, ↓IRF3, ↓IFN ↓CD4^+^, ↓Th17	“CS12192 demonstrated a more selective inhibitory activity on JAK3, as evidenced by decreased activation of p-STATs and p-IRF3, along with IFN down-regulation in cultured cells. CS12192 exhibited dose-dependent amelioration of disease severity, hind body weight loss, paw swelling, and bone destruction in rat models.”	(56)
Nitisinone	OA (Mice with anterior cruciate ligament transection surgery (*ACLT*) -induced osteoarthritis)	Primary chondrocytes (C57BL/6)	In vitro/In vivo	↓cGAS/STING↓NF-κB	“Nitisinone inhibited the cGAS/STING signaling pathway and decreased activation of the STING-dependent NF-κB pathway, thereby reducing extracellular matrix degradation and alleviating chondrocyte inflammation, inhibited osteoclastogenesis and delayed subchondral bone remodeling.”	(20)
Itaconate	OA *(Mice ACLT*-induced osteoarthritis)	RAW264.7	In vitro/In vivo	↑Nrf2/↓STING ↓NF-κB	“Itaconate can activate Nrf2, leading to the inhibition of the STING-dependent NF-κB pathway. Consequently, this alleviates the inflammation, extracellular matrix (ECM) degeneration, and senescence of chondrocytes induced by IL-1β stimulation. Itaconate decreases cartilage degradation and reduces inflammation in the synovial membrane in a mouse model.”	(47)
Auranofin		RAW 264.7	In vitro	↓TBK1, ↓NF-κB, ↓IRF3, ↓IFN inducible protein-10, ↓IFNβ	“Suppression of this TRIF pathway by auranofin coincides with diminished activation of IRF3 and subsequent down-regulation of target genes, including IFNβ and IP-10.”	(57)
Nanodrugs						
Nanomedicine‐in‐hydrogel (NiH): cGAS inhibitor RU.521 and cationic nanoparticles	RA (collagen-induced arthritis in a mouse model)	RAW 264.7	In vitro/In vivo	↓cGAS ↓TNF‐α, ↓IL‐6 ↑Immunosuppressive regulatory T cells (Treg) and ↑myeloid‐derived suppressor cells	“NiH prolongs the lymph node retention of co-delivered agents: the cGAS inhibitor RU.521 and cationic nanoparticles. These components collectively inhibit cGAS pharmacologically and scavenge cell-free DNA (cfDNA), which effectively suppresses proinflammation and reduces the fractions of T helper 17 cells and CD4+ T cells.”	(23)
NiH: polyethyleneimine-coated mesoporous polydopamine nanoparticles loaded with STING antagonist C-176 (PEI-PDA@C-176 NPs)	RA (collagen-induced arthritis in a mouse model)	RAW264.7	In vitro/In vivo	↓STING ↓TBK1, ↓IRF3, ↓IFNβ	“PEI-PDA@C-176 nanoparticles (NPs) were found to significantly diminish joint damage in mouse models of dsDNA-induced arthritis and collagen-induced arthritis by targeting and inhibiting the STING pathway.”	(45)

↓decrease, ↑: increase.

### 3.1 Natural products

The effectiveness of natural products in treating arthritis, such as RA and OA, remains an area of interest and investigation. Natural products, known for their potential anti-inflammatory and antioxidant properties, have been explored for their ability to alleviate arthritis symptoms. These substances may contain bioactive compounds that modulate inflammatory pathways and immune responses (58, 59).

#### 3.1.1 Gelsevirine

Gelsevirine (GS) is an alkaloid that originates from Gelsemium elegans Benth, a traditional Chinese herb. It possesses diverse pharmacological characteristics, encompassing analgesic, anti-inflammatory, and anxiolytic effects. The compound is specifically noted for its robust anxiolytic properties and comparatively lower toxicity when contrasted with other alkaloids (60). Gelsevirine, exhibits potential in relieving OA by specifically targeting the STING pathway. In laboratory tests using chondrocytes stimulated with IL-1β, gelsevirine demonstrates notable effects, such as enhancing cell viability, suppressing matrix-degrading enzymes and pro-inflammatory cytokines, and reducing apoptosis. Importantly, gelsevirine downregulates STING expression in chondrocytes, a factor elevated in OA conditions, contributing to protection against IL-1β-induced damage. In OA mouse models, gelsevirine treatment improves both age-related and surgery-induced OA, evident through lowered OARSI scores, decreased MMP13 positive cells, and increased cartilage area. Notably, the positive outcomes of gelsevirine appear to rely on an intact STING pathway, as observed in STING-deficient mice where gelsevirine fails to provide additional benefits. The proposed mechanism involves gelsevirine facilitating the K48-ubiquitination of STING, indicating its regulatory role in modulating STING activity. Overall, these findings suggest that gelsevirine holds promise as a potential therapeutic agent for OA, acting by mitigating inflammatory and degenerative processes through its modulation of the STING pathway (51).

#### 3.1.2 Catechin

Catechins are polyphenolic compounds found naturally in various plants and foods, with green tea being a notable source. In the context of arthritis, catechins have been investigated for their potential as inhibitors of IKK-β. Catechins, including epicatechin and epigallocatechin gallate, demonstrated inhibitory effects on IKK-β in in vitro assays. In a collagen-induced arthritis mouse model, catechin treatment was associated with reduced paw thickness, clinical scoring, and mRNA levels of inflammatory cytokines (IL-1β, IL-6, and IL-17A). These findings suggest that catechins may modulate the STING pathway, potentially contributing to their anti-arthritic effects. However, further research is necessary to elucidate the detailed mechanisms and therapeutic potential of catechins in arthritis treatment (52).

#### 3.1.3 Glabralactone

Glabralactone, extracted from the Angelica sinensis plant, demonstrates anti-inflammatory effects by effectively suppressing the activation of IRF-3 in LPS-stimulated RAW264.7 cells. IRF-3 is a transcription factor crucial for the innate immune response and cytokine production. In the context of inflammatory diseases such as arthritis and osteoarthritis, glabralactone’s capacity to inhibit IRF-3 activation suggests a potential modulation of the immune response, possibly reducing the expression of proinflammatory agents. Although these findings show promise for glabralactone in alleviating inflammation, further research and clinical investigations are necessary to validate its therapeutic potential in managing inflammatory joint conditions like arthritis and osteoarthritis (53).

#### 3.1.4 Triptolide

Triptolide is a bioactive compound derived from Tripterygium wilfordii Hook F, known for its potent anti-inflammatory properties. Micelles loaded with Triptolide, specifically FA-DexLA@TP (FDL@TP), are designed for targeted drug delivery in the treatment of RA. These micelles, modified with folic acid, aim to actively target M1 macrophages involved in RA-associated inflammation. Administration of Triptolide-loaded micelles, particularly FDL@TP, can down-regulate the cGAS-STING signaling pathway. The modulation of cGAS and STING protein expressions suggests a mechanism by which Triptolide-loaded micelles exert anti-inflammatory effects, making them a potential therapeutic strategy for RA by enhancing drug delivery efficiency and targeting specific immune cells involved in the disease (54).

### 3.2 Synthetic effectors of cGAS/STING

#### 3.2.1 C-176

C-176, a compound recognized for its ability to inhibit the STING pathway, exhibits potential as a treatment for inflammatory osteolytic conditions like RA and OA. Through its impact on the STING pathway, C-176 hinders inflammatory responses and diminishes osteoclast activity, addressing pivotal factors contributing to bone degradation in RA. Experimental models indicate that C-176 provides protection for joint tissues by reducing erosion and enhancing bone parameters. In the case of OA, C-176 enhances subchondral bone microstructure, safeguards cartilage, and diminishes osteoclast activity, resulting in an overall alleviation of OA symptoms. The compound’s mechanism of action involves the modulation of molecular pathways, including NF-κB and MAPK, which are linked to osteoclast differentiation and inflammation. While these findings suggest therapeutic potential, further investigation, including clinical trials, is essential to confirm the safety and effectiveness of C-176 as a viable treatment for RA and OA in human subjects (55).

#### 3.2.2 CS12192

CS12192 is a novel Janus kinase (JAK) inhibitor that selectively targets JAK3, JAK1, and TBK1. JAKs are key signaling molecules in pro-inflammatory cytokine pathways, making them attractive targets for treating autoimmune diseases such as RA. TBK1, involved in type I interferon induction, is also a potential therapeutic target in RA. CS12192 inhibits JAK3, JAK1, and TBK1 activity, reducing the phosphorylation of signal transducer and activator of transcription (STAT) proteins. In preclinical models of arthritis, CS12192 demonstrates significant efficacy in reducing disease severity and hind paw swelling, along with immune modulation effects, including the inhibition of Th17 differentiation and suppression of pro-inflammatory cytokines. These findings suggest that CS12192 holds promise as a therapeutic candidate for RA and other autoimmune conditions, acting through JAK and TBK1 pathways (56).

#### 3.2.3 Nitisinone

Nitisinone (NTBC) is a specific inhibitor of 4-hydroxyphenylpyruvate dioxygenase (4-HPPD), utilized in treating metabolic disorders such as hereditary type I tyrosinemia and alkaptonuria by reducing the accumulation of toxic metabolites. Recent research indicates potential therapeutic effects of NTBC on OA. In chondrocytes, NTBC demonstrates the ability to inhibit TNF-α-induced extracellular matrix degradation, reduce inflammation, and suppress the cGAS-STING/NF-κB pathway. Additionally, NTBC hinders RANKL-induced osteoclastogenesis, crucial for preventing subchondral bone remodeling associated with OA. The study suggests that NTBC acts through multiple mechanisms, including the inhibition of specific cellular pathways, making it a promising candidate for OA treatment. In an *in vivo* OA mouse model induced by anterior cruciate ligament transection (ACLT), NTBC exhibits efficacy in alleviating cartilage degradation and osteoclast differentiation, highlighting its potential as a therapeutic agent for OA (20).

#### 3.2.4 Itaconate

Itaconate, particularly 4-Octyl Itaconate (4-OI), is a derivative of the tricarboxylic acid (TCA) cycle, and it has gained attention for its immunomodulatory properties. Itaconate is implicated in modulating the activity of the STING pathway. STING is a key player in the innate immune response, and its activation leads to inflammatory signaling. The study suggests that 4-OI inhibits the STING-dependent NF-κB pathway, a process associated with inflammation and tissue damage in OA. This inhibitory effect is mediated through the activation of the Nrf2 (Nuclear factor erythroid 2–related factor 2) pathway by itaconate. Nrf2, a transcription factor, regulates genes involved in antioxidant and cytoprotective responses. By activating Nrf2, itaconate suppresses STING-mediated inflammation, extracellular matrix degradation, and cellular senescence in chondrocytes, potentially offering a protective role against OA progression. In addition, itaconate could also alleviate chondrocyte apoptosis induced by M1 macrophages. The findings propose a novel mechanism by which itaconate influences the immune response in OA through STING pathway modulation via the Nrf2 pathway (47).

#### 3.2.5 Auranofin

Auranofin, a gold(I) compound with sulfur-linked organic ligands, is commonly used for RA treatment due to its anti-inflammatory and immunosuppressive properties. It acts by inhibiting NF-κB activation, phosphorylation of JAK1 and STAT3, and the expression of cyclooxygenase-2. This study explores a novel anti-inflammatory mechanism of auranofin, revealing its ability to inhibit TLR4 dimerization induced by lipopolysaccharide (LPS). As dysregulated Toll-like receptor (TLR) activation is implicated in chronic inflammatory diseases like RA, the study aims to identify auranofin’s molecular target in the TRIF-dependent pathway of TLR3. The findings indicate that auranofin suppresses poly[I:C]-induced NF-κB and IRF3 activation, phosphorylation of IRF3, pinpointing TBK1 as the molecular target. This suggests that auranofin may have therapeutic potential for dampening inflammatory responses associated with TLR activation, specifically through its action on TBK1 in the TRIF-dependent signaling pathway of TLRs (57).

### 3.3 Nanodrugs

Nanomedicine-in-hydrogel (NiH) (61) emerges as a promising therapeutic approach against RA. This innovative strategy involves the formulation of cationic nanoparticles (cNPs) with cfDNA-scavenging properties and a cGAS inhibitor (RU.521) into an injectable hydrogel. NiH is designed to target lymph nodes (LNs), particularly addressing the enlarged LNs associated with RA. Upon subcutaneous injection, NiH efficiently homes in on LNs, where it acts to scavenge cell-free DNA (cfDNA), inhibit the cGAS-STING pathway, and modulate the immune microenvironment. By repolarizing macrophages, expanding regulatory T cells (Treg) and myeloid-derived suppressor cells (MDSCs), and reducing inflammatory cell populations, NiH aims to induce systemic immune suppression, promoting an anti-inflammatory state and restraining RA progression. The hydrogel component enables controlled drug release and targeted delivery, ensuring sustained therapeutic effects while minimizing systemic dissemination. NiH represents a targeted and multifaceted nanomedicine strategy to address the underlying molecular mechanisms of RA and modulate the immune response for therapeutic benefits (23). Similarly, another research has investigated the potential of NiH approach, specifically using polyethyleneimine (PEI)-coated polydopamine (PDA) nanoparticles loaded with the STING pathway inhibitor C-176, for the treatment of RA. The study begins by highlighting the role of the STING pathway in inflammatory diseases, emphasizing its dysregulation in RA. The researchers hypothesized that cationic nanoparticles (NPs), particularly PEI-modified NPs, can scavenge double-stranded DNA (dsDNA) associated with RA pathology, thereby reducing STING activation. The NiH, composed of PEI-PDA@C-176 NPs, was designed for local delivery and sustained release, aiming to inhibit the STING pathway and alleviate inflammation in RA. The accumulation of cell-free dsDNA and activation of the STING pathway in RA synovium and a mouse model of RA was observed. Genetic deletion of STING was shown to ameliorate RA pathogenesis. The NiH strategy was implemented by developing and characterizing PEI-PDA@C-176 NPs, which exhibit good biocompatibility. In vitro experiments reveal the NPs’ ability to inhibit STING activation and dsDNA-induced inflammation. The therapeutic efficacy of PEI-PDA@C-176 NPs is then demonstrated in both dsDNA-induced arthritis and the collagen-induced arthritis model, showing a reduction in clinical scores, synovial hyperplasia, cartilage destruction, and bone damage. The study suggests that PEI-PDA@C-176 NPs exert their effects through the STING pathway, offering a potential targeted and multifaceted nanomedicine strategy for treating RA (45).

## 4 Conclusion

The cGAS-STING signaling pathway, responsible for sensing cytoplasmic DNA and triggering inflammatory responses, and its downstream targets TBK1, IRF-3, and IKK play a crucial role in the progression of RA and OA. In RA, the cGAS-STING pathway is central to aggressive synovial tissue behavior, while in OA, it is triggered by specific fragments, impacting cartilage breakdown. cGAS, STING, and TBK1 are implicated in RA, influencing inflammation and immune responses. STING’s role is intricate, possibly acting as a negative regulator in early RA stages. TBK1 is crucial for sustaining inflammation in both RA and OA, with potential therapeutic implications. Elevated IRF-3 and IKK in RA synovial tissue contribute to immune responses. The findings underscore the intricate nature of these pathways in arthritis development, suggesting potential targets for therapeutic intervention. Targeting the cGAS-STING pathway is a therapeutic intervention against OA and RA. The cGAS-STING pathway, responsible for sensing cytoplasmic DNA and triggering inflammatory responses, plays a crucial role in the progression of these joint disorders. Various pharmaceutical approaches, particularly natural products and synthetic effectors, can target this pathway. Examples include Gelsevirine, a compound from a traditional Chinese herb, which shows promise in relieving OA by modulating the STING pathway. Catechins, Glabralactone, Triptolide, and Auranofin are explored for their potential anti-arthritic effects through different mechanisms. Additionally, nanodrugs, specifically Nanomedicine-in-hydrogel, can be used as a targeted approach to inhibit the cGAS-STING pathway in RA, aiming to induce systemic immune suppression and restrain disease progression. These approaches present diverse strategies for managing OA and RA by addressing the underlying molecular mechanisms of inflammation and immune response.

## Author contributions

XY: Writing – original draft, Writing – review & editing. LZ: Writing – original draft, Writing – review & editing. YP: Writing – original draft, Writing – review & editing.
